# What Do We Know about Young Volunteers? An Exploratory Study of Participation in Zooniverse

**DOI:** 10.5334/cstp.248

**Published:** 2020-01-13

**Authors:** Christothea Herodotou, Maria Aristeidou, Grant Miller, Heidi Ballard, Lucy Robinson

**Affiliations:** * The Open University, GB; † University of Oxford, GB; ‡ University of California Davis, US; § Natural History Museum London, GB

**Keywords:** Citizen Science, young people, online participation, Zooniverse

## Abstract

Citizen Science (CS) is an increasingly popular activity enacted either in the field or online. Volunteers participate in research activities such as data processing and analysis by, for example, identifying plants and animals. In this paper we examine young people’s participation in online CS projects hosted on the Zooniverse platform. This is an exploratory study, the first of its kind that focuses on young people, mainly 16−19 years old. It uses data analytics and visualisation techniques to capture participation in online CS, and in particular to answer the following questions: (a) What does young people’s participation look like in CS projects? (b) What Zooniverse projects do young people choose to participate in? and (3) What Zooniverse projects do young people choose together? Findings revealed five distinct engagement profiles characterising young people’s participation and identified certain projects as been more popular across participants. Implications for the design of online citizen science projects targeting young people are discussed.

## Introduction

Citizen science (CS) refers to the participation of the general public or volunteers in research activities such as data collection and analysis. It is an increasingly popular activity enacted either in the field or online. An example of a field-based CS program is bioblitzes; these are short-term events lasting usually for a day, during which volunteers are asked to find and photograph as many species (plants, animals) as possible within a set location and time (Robinson et al. 2013). A significant number of projects are also hosted virtually or have online components (Kloetzer et al. 2013). In these projects, participants are asked to complete online tasks such as identifying living species or classifying galaxies on platforms such as Zooniverse (zooniverse.org) and iNaturalist (inaturalist.org). These activities are of benefit to both scientists and volunteers; scientists conduct time-consuming and expensive projects that could not be done without the support of thousands of volunteers, and volunteers may gain a better understanding of science and the scientific method, appreciate nature, and support conservation initiatives (Freitag and Pfeffer 2013). CS programmes are often viewed as opportunities for educating the general public and opening up science and the work of scientists to volunteers (Bonney et al. 2016; Herodotou, Sharples, and Scanlon 2018).

Specific groups of people are found to predominantly populate CS field-based programmes. These are mainly white, middle-aged (30−60), scientifically educated males with an interest in science (Brossard et al. 2005; Price and Lee 2013). For online CS programmes, participation patterns are also skewed; a minor number of adult volunteers makes the great majority of contributions (90%) over time (Eveleigh et al. 2013), while the majority of volunteers contributes only once (Ponciano et al. 2014). Pre-existing interest in science, software, and community aspects are amongst the factors motivating initial participation, whereas factors such as recognition, gaming elements, team-play, and interest in the topic are found to sustain participation (Aristeidou et al. 2014). However, all these findings are focused on adults or do not distinguish age groups; our understanding of young people’s participation in online CS programmes is entirely lacking. These insights raise the need to capture and understand online CS engagement patterns and project choices. Such knowledge can inform the design of online CS projects as well as the implementation and assessment of new engagement strategies that can invite more diverse audiences to participate in CS. The redesign of existing CS programmes or the design of new ones targeting specific audiences can widen participation and reach out or retain groups of volunteers not currently engaged in CS activities.

In this paper, we focus on a specific sub-population, young people 5−19 years old who participate in online CS projects hosted on the Zooniverse platform. To the best of the authors’ knowledge, no studies have yet been conducted examining young people’s participation in online CS. Anecdotal evidence suggest that young people (less than 18 years old) have joined projects in Zooniverse (see Zooniverse blog, 2015), yet their participation has not been analysed in any follow-up examinations. Other evidence suggests that young people make use of mobile technologies in field-based CS programs, such as the after school “Science Action Club” that engages school children with field-based CS activities in which they capture and share observations using the mobile application and online platform iNaturalist (iNaturalist.org). Researching young people in informal settings entails certain challenges such as identifying and recruiting young people and gaining or confirming consent with guardians. Yet, as arguments increase for the learning potential of online CS programmes for young people in particular, understanding their participation can provide insights about, for example, the duration and intensity of their engagement, their loyalty to CS activities, and the projects they join more or less often. Such data can inform the design of CS projects targeting young people, support international efforts to engage them with STEM topics, and raise their interest and curiosity in scientific careers.

The aim of this study is to explore and characterise young people’s participation in online CS projects and to discuss implications for the design of online CS projects that target young people. We make use of data analytics and visualisations, clustering, and Social Network Analysis (SNA) techniques to understand participation of young people in one of the larger web-based citizen science platforms, Zooniverse, currently hosting more than 50 active projects and 1.6 million registered users. Our specific research questions (RQs) are:

(1) What does young people’s participation look like in CS projects?
(2) What Zooniverse projects do young people choose to participate in?
(3) What Zooniverse projects do young people choose together?

This study is part of the LEARN Citizen Science project (https://education.ucdavis.edu/ccs-learn-citsci), an international research collaboration among three Natural History Museums (NHMs) and three research institutions in the UK and US that aims to capture participation and learning in online and field-based CS settings and to improve the design of CS programmes offered by NHMs. This includes the partnership between Zooniverse and the NHMs on projects that leverage the crowdsourcing platform to answer biological research questions of the NHMs. In the next sections, we present literature about CS participation and how data analytics can be a useful methodology for understanding participation and learning in informal science settings.

## Background Literature

### Participation in CS programmes

Participation takes multiple forms in CS programmes and refers to either the level of volunteers’ involvement in scientific activities or the type of activities and intellectual effort needed. Shirk et al. (2012) classified projects based on the level of volunteers’ *involvement in scientific activities* into: (a) contractual projects initiated by scientists to address a community need, (b) contributory projects designed by scientists in which the public collects data, (c) collaborative projects in which the public, in addition to collecting data, refines the project design, analysis, and dissemination, (d) co-created projects designed in collaboration with the public, and (e) collegial contributions initiated by non-professional members of the public who conduct research independently.

For online CS programmes in particular, Ponciano et al. (2014) classified participation in two Zooniverse projects (Galaxy Zoo, Milky Way) based on levels of engagement of volunteers into *transient* and *regular*. Transient users completed tasks only once and did not return to the platform again. They participated sporadically in CS activities, most likely due to a help request made by others. In contrast, regular users returned to the platform at least once to complete more tasks. They could be described as volunteers who actively seek opportunities for participation in CS projects. Online participation was found to be mainly transient, yet regular volunteers completed the largest number of tasks. Similarly, Eveleigh et al. (2014) described participation in terms of *high* and *low contributions*, with the latter referring to small input and little involvement and the former to regular and significant participation, including social participation in forums. In a follow-up analysis, Ponciano and Brasileiro (2014) proposed five engagement profiles: Hardworking (hard work, yet leaving the project soon), spasmodic (short period of contributions with irregular periodicity), persistent (link to the project for long, yet with few active days), lasting (similar to persistent profile, yet remaining linked to a project for a shorter time period), and moderate (the shorter the period of time volunteers are linked to a project, the more days they come back to the project to complete tasks). Volunteers were found mainly in the moderate profile with a minority in the persistent profile. Lurking members also were detected in CS programmes, indicating non-active participation (no contributions) (Aristeidou et al. 2017).

In the broader Human-Computer Interaction (HCI) literature, participation, referred to as “engagement,” has been classified on a continuum based on the *type of activities* that users engage with and the *intellectual contribution* required. Preece and Shneiderman (2009) describe online participation as initiated by *reading* activities, developed into *contributing* data in the form of a question or a picture, extended to *collaborative* activities such as an article creation in Wikipedia, and reaching *leading* activities, for example, mentoring novices. The number of participants decreases from reading to leading activities. Similarly, Haythornthwaite and De Laat (2010) classified online activities as *lightweight* (simple, often directed by others) or *heavyweight* (complex and time consuming, such as academic writing).

In the case of Zooniverse, projects are more relevant to *contributory* forms of participation as described by Shirk et al. (2012), yet the public is processing, rather than collecting data, as described by Bonney et al. (2016), where data are originally collated and presented in the platform by scientists. In terms of the types of data processing activities that Zooniverse hosts, we categorised these into (a) “tasks requesting a response to a question” such as “Are there any penguins in this image?” in the *Penguin Watch* project; (b) “free text entry tasks” such as “Add keywords to describe each illustration” in the *Science Gossip* project; (c) “marking tasks” such as *Project Plumage* requesting to mark-up different views of bird specimens; and (d) “identification tasks” such as those found in the majority of camera trap projects on Zooniverse (e.g., *Camera CATalogue*), which ask volunteers to identify the species they can see from a set list. What determines effort or intellectual contribution is how easy or difficult the question being asked in each project is as well as the series of tasks (and accompanied time devotion) participants are asked to address within each project. An easy task could be a binary question, such as the first task on the *Penguin Watch* project, which asks: “Are there any penguins in this image?” Most people can recognise a penguin and answer “yes” or “no” when they see one. On the contrary, the yes/no question “Are there Meridiani-type polygonal ridges visible in this image?” in the *Planet Four: Ridges* project is rather complex, due to lack of familiarity with the scientific terms and the data in question for a majority of people. An example of a time-consuming task is the second task on the *Penguin Watch* project, which requests participants to mark the locations of all the penguins in a given image; although identifying penguins is a relatively easy activity, in some cases hundreds of penguins should be marked in a single image. In this study, we examined (as part of RQ2) whether and how different task types relate to participation patterns, as a means to understand whether projects with, for example, “easy” or less time-consuming tasks are more or less often chosen by young people.

### Capturing participation in informal learning settings

Learning Analytics (LA) typically refer to data generated from learners’ interaction with, for example, a Learning Management System (LMS) and their application to improving teaching and learning. LA have been used less frequently to understand participation and learning in informal learning settings (Ferguson et al. 2016). Such studies have examined learning at the workplace (e.g., Littlejohn 2017), interactions in technology-mediated social systems such as online forums (e.g., Kloetzer, Schneider, and Costa 2016), and a few CS projects examining adult participation (Aristeidou et al. 2017; Dittus, Quattrone, and Capra 2016; Morais, Santos, and Raddick 2015; Ponciano and Brasileiro 2014). In this paper, we use LA as a methodology that allows a fine-grained analysis of young people’s participation (aged 5−19) in Zooniverse projects as a first step towards understanding and improving their learning experiences in online CS. We perceive participation as a requirement of learning; understanding how young people participate in CS programmes will enable us to identify how or what types of participation relate to, facilitate, or hinder learning processes and outcomes and improve the design of CS projects accordingly.

Also, we perceive online CS projects as “informal learning settings,” as these have not been designed with an explicit learning or curriculum objective, learning is rather random and spontaneous, and is less likely to lead to any form of recognition.

CS studies that have made use of LA propose or develop a set of metrics for capturing online participation for adult volunteers. Amsha et al. (2016) explicitly proposed the use of LA for understanding learning and participation in online CS programmes. They introduced and tested a non-exhaustive set of indicators for demographic, learning, engagement, creativity, and collaboration related LA, such as total number of tasks over the activity duration (engagement-related LA), average percentage of tutorial completion (learning-related LA), length of blog posts (creativity-related LA), and number of artefacts shared (collaboration-related LA). Ponciano and Brasileiro (2014) captured Zooniverse engagement over time considering points of engagement, periods of sustained engagement, disengagement, and re-engagement. Their metrics (adopted also in this study) capture time potentially linked to a project, days actually linked to a project, active days, time devoted to tasks per day, and elapsed time between two active days. Morais, Santos, and Raddick (2015) highlighted the evolution in number of volunteers and classifications over time and showed that the majority of classifications in Zooniverse were completed in the first 600 days of the project, and sharply declined afterwards. They also explained “bursts” of activity and analysed volunteers joining or leaving a project in short intervals. They cluster volunteers into *committed, potential*, and *curious* based on the following metrics: Relative duration activity, frequency of activity per day, and classifications completed within a single day.

Dittus, Quattrone, and Capra (2016) compared the design of three humanitarian mapping projects using cohort and task analysis and first-time contributor’s performance. Projects designed as sustained initiatives that engage volunteers through various means, such as social media and mapathons, exhibited high retention rates. In contrast, projects with complex tasks were found to demotivate first time contributors. Improvements in performance were not related to increased retention. Aristeidou et al. (2017) examined participation of volunteers in an online community where participants could not only contribute to CS projects but also initiate and pursue their own CS activities. They expanded the Ponciano and Brasileiro (2014) engagement metrics by proposing the lurking ratio, that is, the proportion of days that a participant was lurking (logging in a platform, browsing, yet not contributing) in relation to the total days that they visited the project.

## Methodology

### Process of recruitment and consent

Aligning with the broader LEARN Citizen Science project objectives, we aimed to identify and examine the participation of at least 100 volunteer young people (ages 5−19) on Zooniverse projects that are related to the Natural History Museum (NHM) of London, UK. This number target was rather arbitrary given the lack of any previous examinations referring to the numbers of young people possibly joining online projects that could inform our study. The only NHM active project at the time was *Project Plumage*, asking participants to mark-up photos of different coloured birds. This activity aimed to help scientists understand feather colour evolution over time. Due to the low number of participants in the project (about 2,000 at the time) and to increase the number of volunteers, we ran various promotional campaigns (emails, newsletters to Zooniverse volunteers) originally raising awareness only for *Project Plumage*. The marketing campaign lasted for two and a half months. This approach resulted in increasing the overall number of registered volunteers to around 3,000, yet the number of young people attracted through this publicity did not meet our initial target of 100 young people. This resulted in expanding the scope of the study to other Zooniverse projects that focused on NHM-related research questions or collections, including *Science Gossip, Notes from Nature* (participants transcribe photos of specimens involving many NHM digitized collections), *London Bird Records* (participants transcribe historical records of birds), *Orchid Observers*, and *Penguin Watch*. This follow-up campaign lasted for two months.

Users interested in the study followed an email weblink. An information sheet briefed potential participants about the aim and procedures of the study, and an online form invited them to consent in participating (if over 16 years old), or to provide their guardians’ contact details (if younger than 16 years old) for researchers to confirm consent over the phone with guardians. Although 159 young people originally signed up for the study, the final number was 104, as 22 of them were duplicates or their usernames did not exist on the Zooniverse database, 19 did not provide consent, and 14 did not make any contribution to the platform. Log data extracted from the platform for analysis were anonymised, that is, Zooniverse usernames were changed to, e.g., User1, User 2. The study received ethical clearance from the university of the lead author.

### Sample characteristics

The great majority of the sample were female (67%, n = 70), 29% were male, three users selected “other,” and one preferred not to disclose gender information (total n = 104). In contrast to existing CS projects and the predominance of male adults in CS activities (Brossard et al. 2005; Price and Lee 2013), young participants were found to be mainly female. This insight warranted further investigation and resulted in examining gender differences in RQ1, in particular comparing the number of classifications made between male and female and RQ2, comparing the gender distribution in the choice of CS projects in which young people participate. In terms of age, a great majority (n = 99) was aged 16−19, two users were aged 13−15, two users were aged 10−12, and one user was under 10 years old. In terms of the device used to access Zooniverse, a great majority (n = 76) used a desktop computer, 16 used a mobile device, and 11 used both devices.

### Process of data analysis

#### Online metrics

To measure online engagement, we adopted the engagement metrics of Ponciano and Brasileiro (2014), which were used to describe adult participation in two Zooniverse projects. This is the only study with a corresponding data-set that would allow us to make comparisons between the engagement patterns of adults and young people in Zooniverse. Yet, we note a large difference in the sample size and the Zooniverse projects under examination (i.e., 104 young people across all Zooniverse projects in our study versus 6,093 in The Milky Way project and 23,547 in Galaxy Zoo). These metrics were combined to develop engagement profiles (clusters) grouping young people with similar or different patterns of behaviour together.

a) *Activity ratio* is the ratio of days on which a user was active and contributed at least one classification in relation to the total days they remained linked to Zooniverse. The closer to one, the more active a user is during the days they are linked to the project.
b) *Relative activity duration* is the ratio of days during which a user is linked to the project to the total number of days from their Zooniverse registration to the date that Zooniverse data were aggregated for this study (30th August 2018). The closer to one, the longer a user remains linked to the project.
c) *Variation in periodicity* is the standard deviation of the multiset of number of days elapsed between each pair of sequential active days. The closer to zero, the steadier the rate by which a user returns actively to Zooniverse. For example, if a young participant visited Zooniverse on the 3rd, 4th, 10th, 20th, and 28th of June, then the multiset on which the standard deviation is applied to is {1, 6, 10, 8}.
d) *Daily devoted time* is the average hours that a user spends on Zooniverse tasks per day they are active.

#### Clustering analysis

Cluster analysis, performed with the statistical package SPSS, was used to determine engagement profiles amongst participants and to answer RQ1. The engagement profiles characterise the level of engagement of users that belong to the specific profile. Engagement profiles were created according to the metric results. The values calculated for each user in the metrics were first normalised in the interval of [0, 1]. Prior to clustering, users were first separated into two groups: Those who had more than two active days, and those who had two or fewer active days. As it was impossible to calculate a “variation in periodicity” value for the second group, this was excluded from the initial clustering analysis of the first group.

**Figure 1:**
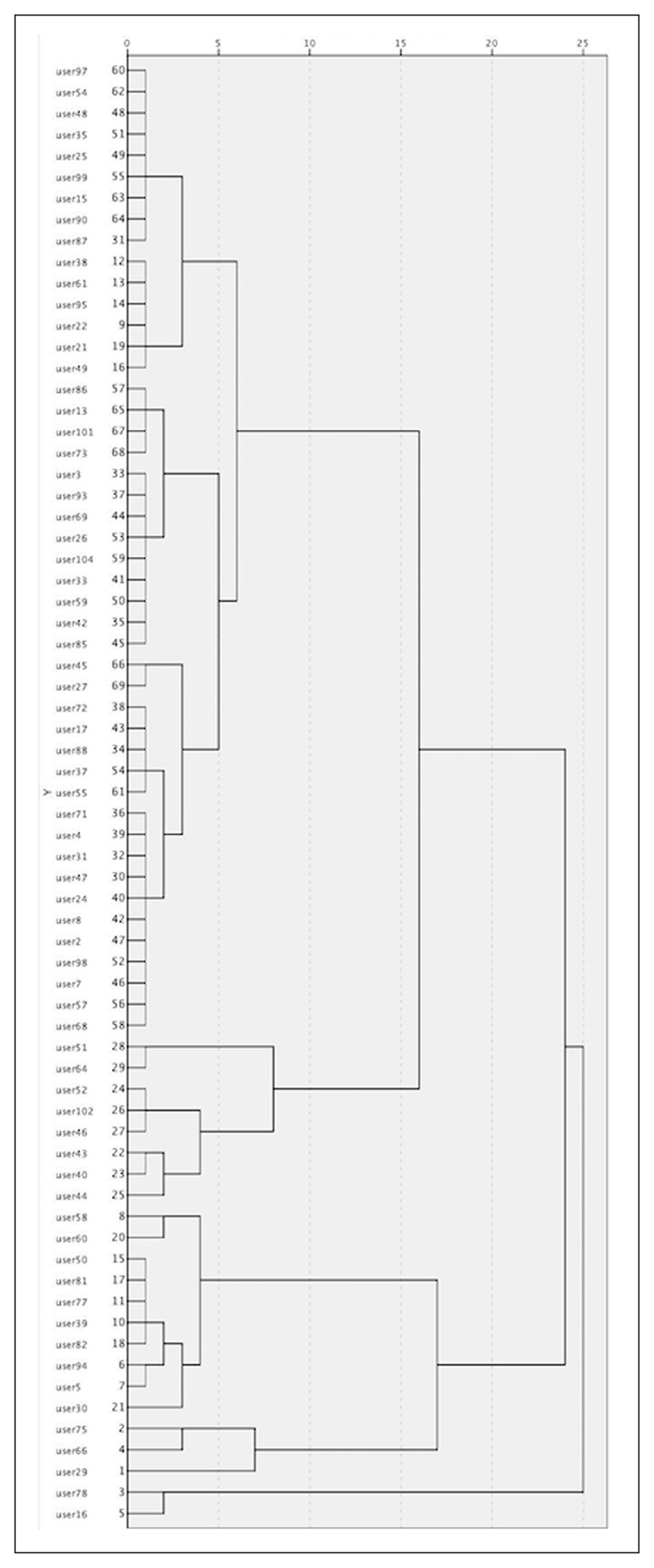
Dendrogram showing the participants of the study (the “visitors” category is excluded).

Dendrograms for both groups were plotted using a hierarchical agglomerative clustering algorithm (Figure 1), to provide suitable intervals to test the number of clusters for each engagement profile. The dendrograms displayed the distances at which users and clusters are joined on a scale of [0, 25]. Vertical lines on the dendrogram helped in counting the potential number of clusters by counting the number of lines they intersect. A one-way ANOVA test for the first clustering outcomes showed that the *p*-value for the “daily devoted time” metric was very high in all the potential numbers of clusters, therefore the variable was not significantly different between the clusters. This resulted in excluding this metric from the clustering analysis. The clustering quality was then evaluated by comparing the within group sum of squares and the between sum of squares for each potential number of clusters (Anderberg 1973). The within group sum of squares measured the differences between the users and the centre of the group to which they belong, while the between groups sum of squares measured the differences among the group means. The best clustering scheme is the one that minimises the within group sum of squares (intra-cluster similarities) while maximizing the between group sum of squares (inter-cluster dissimilarities). The *K*-means algorithm was then used to classify the data with the number of clusters found through the within and between sum of squares. The classification results of each cluster were visualised through box-plots and comparative bar charts.

#### Social Network Analysis (SNA)

We used Social Network Analysis (SNA) to analyse young people’s choices of Zooniverse projects and to identify which projects tend to be chosen together (RQ3). The rationale behind RQ3 was to identify whether young people tend to choose and join projects that share certain common characteristics (e.g., camera trap projects) and therefore express interest in specific research areas. Such insights could suggest which projects should be offered to effectively engage young people with CS activities. SNA conceptualises people or resources as nodes, connected by ties, when a link exists between two nodes. Zooniverse projects were represented as nodes in a graph. Ties between nodes demonstrated co-chosen projects. Contributions from all participants were added to the network. The lists of projects that each participant contributed to were then rearranged to make pairs of co-chosen projects. The undirected ties that link two projects show that a participant contributed to both of these projects. Duplicates were allowed to produce a weighted graph and show the importance of the link between projects. This method has been used for co-joined citizen inquiry missions (Aristeidou et al. 2017) and co-studied massive open online courses (Jordan 2014). A spreadsheet with data was imported into the Gephi visualisation tool producing an undirected network. The social network graph demonstrated the betweenness centrality of Zooniverse projects, that is, the projects that are most interconnected with other projects (they are co-chosen along with other projects). The network comprised 148 nodes and 9,306 ties. The project interconnection was then explored, detecting the most and least popular projects. A centrality degree metric showed the most chosen projects. A modularity algorithm detected groups of projects that tend to be chosen together. Colouring the different groups helped in understanding whether participants were interested in particular projects or were open to take part in any project.

#### Statistical comparisons between groups

A test of normality and inspection of the Q-Q plots revealed that the data were not normally distributed (*p* < .001). Therefore, independent Kruskal-Wallis tests were performed to compare male and female participation, in particular to identify whether there are significant differences in (a) the number of contributions (classifications) made between males and females and (b) whether males and females choose to participate in specific projects.

## Results

### Engagement metrics

The four engagement metrics were compared to adult Zooniverse users (see Ponciano and Brasileiro 2014). Young participants, engagement metrics (a) *M* = 0.31, *SD* = 0.44; (b) *M* = 0.02, *SD* = 0.11; (c) *M* = 0.49, *SD* = 0.36; (d) *M* = 49.93, *SD* = 52.08 were found to be relatively less active than adults during the days they are linked to the platform and to devote considerably less time daily on Zooniverse tasks. Compared to adults, engagement metrics (a) *M* = 0.40, *SD* = 0.40; (b) *M* = 0.44, *SD* = 0.54; (c) *M* = 0.20, *SD* = 0.30; (d) *M* = 18.27, *SD* = 43.31, they were found to be linked to the platform for a longer period of time, and they were less systematic in their visits.

### Engagement profiles

**Figure 2:**
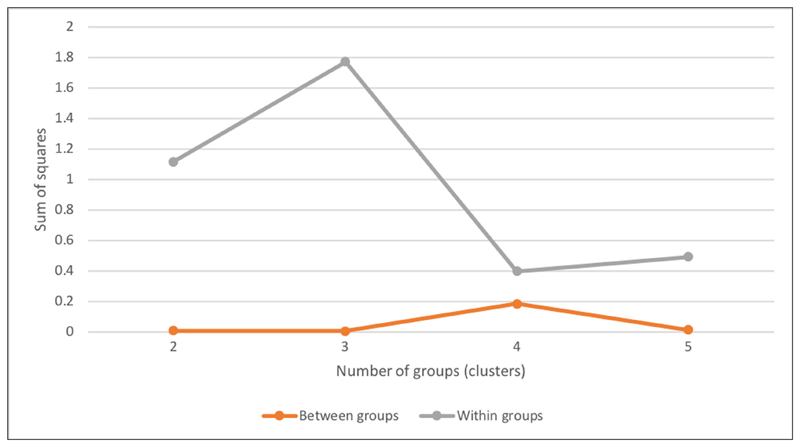
Similarity between clusters (bottom line) and within clusters (top line).

**Figure 3:**
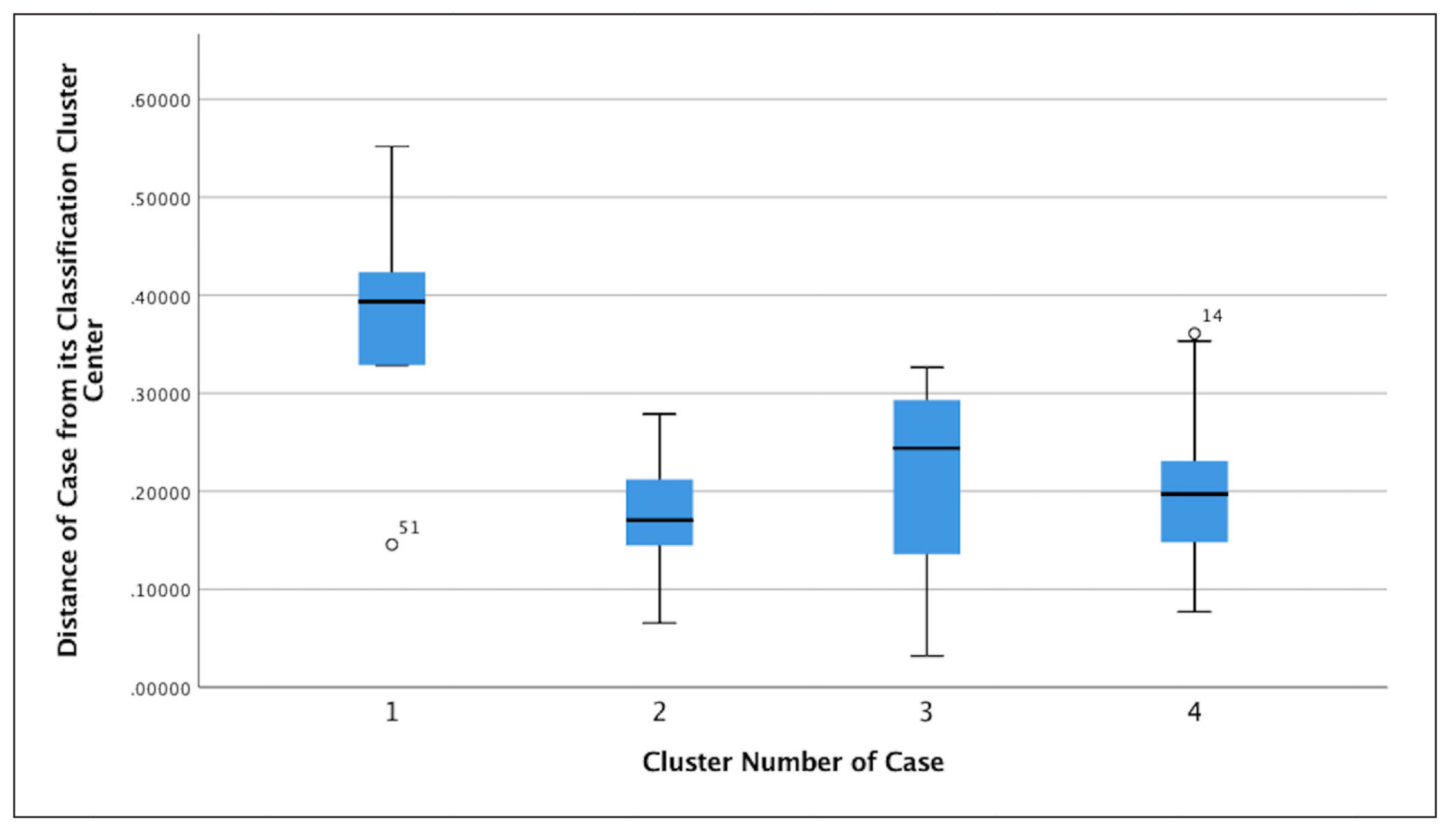
Box-plots presenting differences amongst the four clusters. (Note: “51” indicates one participant who did not fit in any of the clusters.)

**Figure 4:**
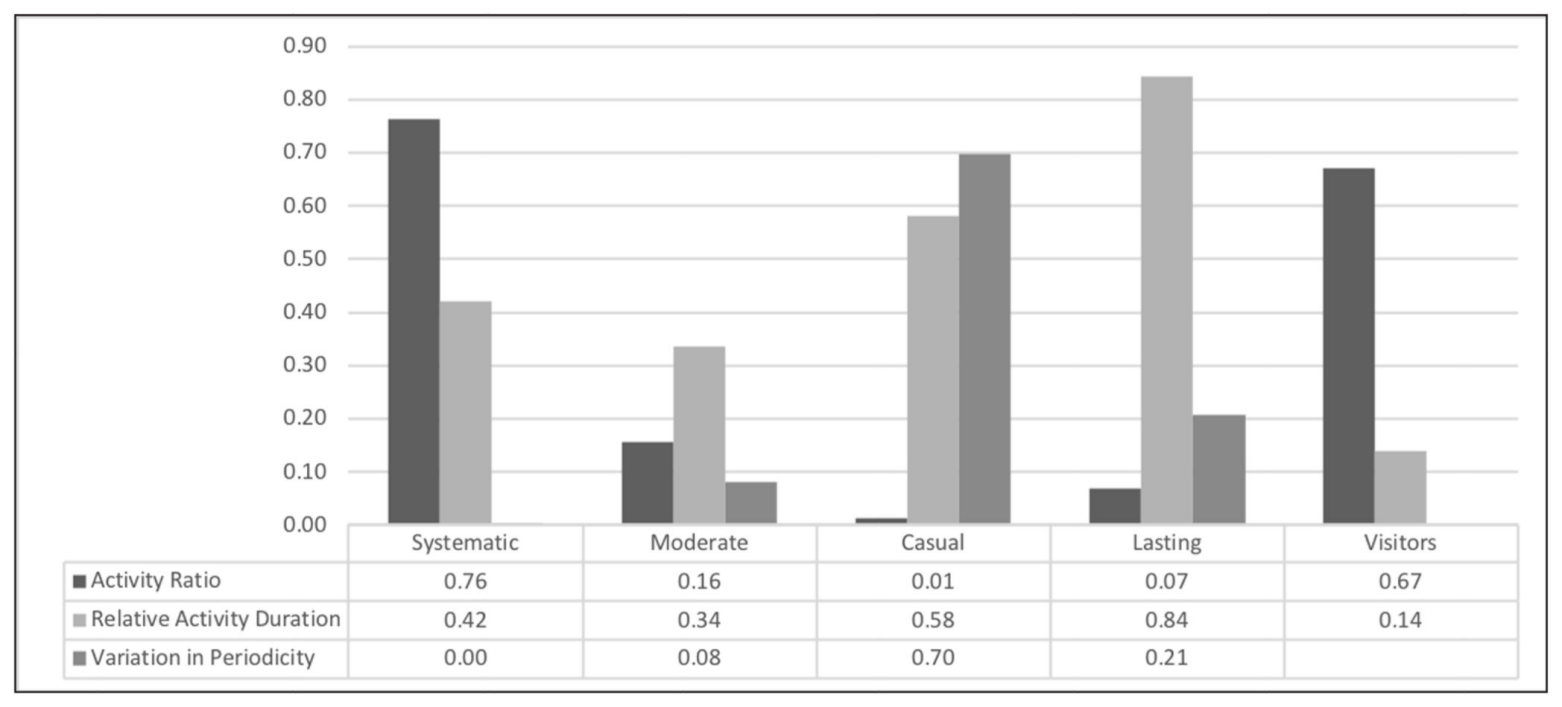
Engagement profiles of young people in Zooniverse.

Participants with two or fewer active days on the platform formed a separate profile labelled as “visitors.” The rest of the participants were clustered according to the metrics of Ponciano and Brasileiro (2014) (“daily devoted time” was excluded, see previous sections). The hierarchical clustering algorithm, with the dendrogram cut-off vertical lines at the scales values of “25,” “20,” and “15” (Figure 1), indicated between two and five clusters as the interval to be tested, suggesting that four clusters is the optimal solution in terms of number of clusters (Figure 2). An overview of box-plots (Figure 3) revealed an outlier far away from the lower whisker of cluster 1, and this was excluded from the dataset. The *K*-means algorithm was used to classify the data into four clusters. Clusters were labelled based on the core activity of the cluster (Figure 4). In two cases, labels were adopted from the study of adult Zooniverse users.

#### Cluster 1: Systematic engagement (n = 5)

This category demonstrates the largest activity ratio, combined with moderate relative activity duration and zero variation in periodicity. Young people in this category visited the platform at regular time intervals and were very active during their stay on Zooniverse. However, the moderate relative activity duration showed that compared to some other profiles, these participants did not remain linked to Zooniverse for a long time. Young people in this category were found to be all female, aged 16−19, who contributed from 257 to 1,625 classifications to 9−33 projects. One participant shared her account with her family. A representative of this category is User 29; she registered with Zooniverse on the 7th of August and contributed 420 classifications to 19 projects, on the 7th, 8th, 9th, and 10th of August.

#### Cluster 2: Moderate engagement (n = 16)

Members of this category exhibited moderate relative activity duration, low activity ratio, and low variation in periodicity. The latter shows that their visits were at a constant rate, yet they were neither linked to the platform for a very long time nor very active during that period. This category consisted of 11 female and five male users, who did not share their accounts with others (e.g., family, friends). Only one member was younger than 10 years old; the rest of the participants were aged 16−19. Members of this category contributed between 62 and 3,171 classifications to 1−25 projects. An example, User 94 (female, 16−19 years old), registered on the 29th of June 2018 and contributed 62 classifications to three projects on the 29th of June and 12th and 19th of July.

#### Cluster 3: Casual engagement (n = 8)

Members of this cluster were characterised by high variation in periodicity and relative activity duration, while they had extremely low activity ratio levels. Although they remained linked to Zooniverse for a long time, they had inconstant visits and were not very active during the period that they were linked to the platform. They were five female and three male participants, aged 16−19, who did not share their accounts with other people. They contributed from 65 to 651 classifications to 2−27 projects. An example, User 43 was female and registered in March 2013. She contributed 635 classifications to 27 projects. Although registered in 2013, she made her first classification on the 21st of July 2015, followed by more classifications in the following months.

#### Cluster 4: Lasting engagement (n = 40)

This category is characterised by the largest relative activity duration, low variation in periodicity, and low activity ratio. Members of this category remained linked to Zooniverse the longest, did not visit the platform very regularly, and had a small number of active days during the long period that they were linked to Zooniverse. The majority of young people are found in this cluster. This category consists of 28 female, nine male, and three who declared themselves as “other.” Apart from two members, the rest were aged 16−19. One member was aged 13−15, and another was aged 10−12 and shared her account with her mother. Members of this cluster contributed from 38 to 4,558 classifications to 3−42 projects. An example, User 47 was female, 16−19 years old, and registered on the 8th of March 2018. She had only three active days since registration, during which she contributed 449 classifications to five projects.

#### Cluster 5: Visitors (n = 34)

Members of this cluster contributed to projects one or two days only, and therefore their variation in periodicity could not be calculated. Their activity ratio is nearly as large as that of Cluster 1, but this was accompanied by low relative activity duration, indicating active days during their short stay in Zooniverse. Metric values in this cluster and the dendrogram suggested two sub-groups of this category. The first subgroup (n = 12) was linked to the project for longer than the second, but it was active only one or two days during this time period. It consisted of eight female and four male users, all aged 16−19, with none sharing their account with others. Nine members contributed to two projects only, while one user contributed to 16 projects. For example, User 79 (female, 16−19 years old) registered with the platform in April 2016, and had only two active days since then, in December 2016 and July 2017. She made 28 contributions to two projects. The second subgroup (n = 22) was linked to the project for a maximum of two days and was very active during those days. The group included 13 female users, eight male and one participant who did not disclosed their gender. All but two participants were aged 16−19; one was aged 13−15 and one was aged 10−12. One participant, who was aged 16−19, shared her account with family and friends. Although the majority of this category (n = 13) contributed to a single project only, there were users who contributed to as many as ten projects. An example, User 34 had a single active day. She was female, 16−19 years old, and registered on 29 June 2018. She contributed 64 classification to a single project, *Project Plumage*.

**Figure 5:**
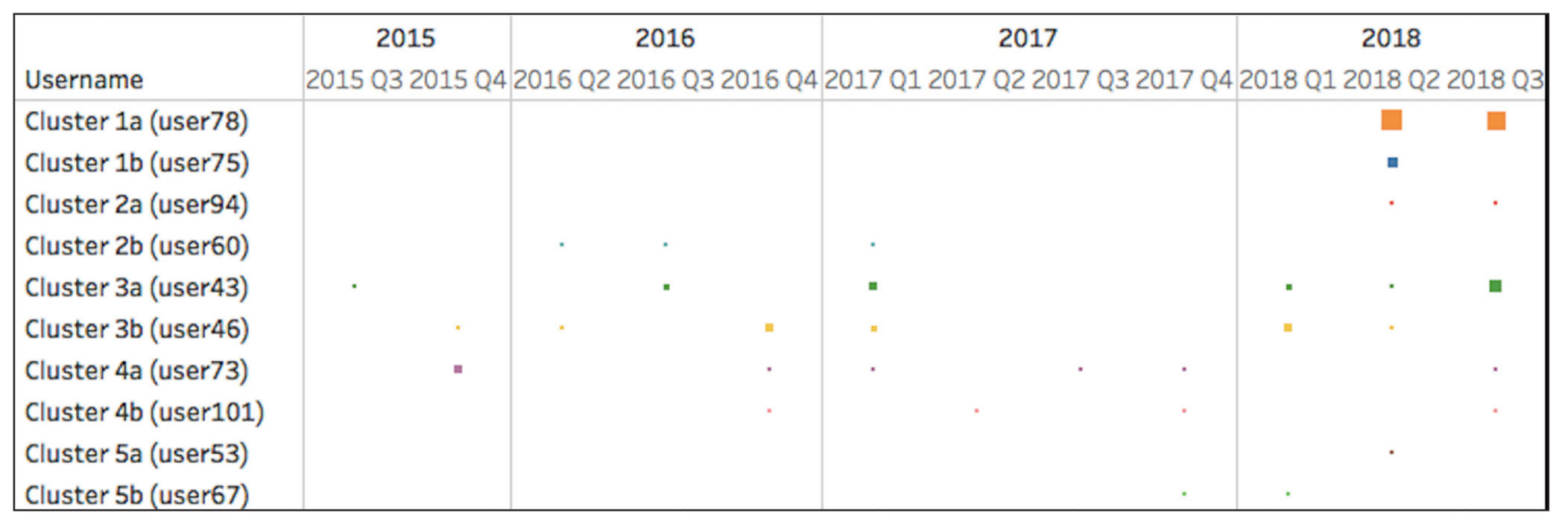
Examples of participants from each cluster (Q = Quarter of a year; e.g., 2015 Q3 = third quarter of 2015).

Figure 5 shows examples of how participation changed over time based on year quarters (e.g., 2015 Q3: third quarter of 2015) for specific users from each engagement profile. For example, User 75 was a systematic user (Cluster 1), and all of their activity was recorded in the second quarter of 2018. User 60 (Cluster 2) visited the platform regularly between the second quarter of 2016 and the first quarter of 2017, yet they did only a few tasks on the platform. User 46 (Cluster 3) had visits on the platform over a long period of time (between 2015 and 2018), but these visits were rather random and accompanied by low activity.

### Frequency of contributions

**Figure 6:**
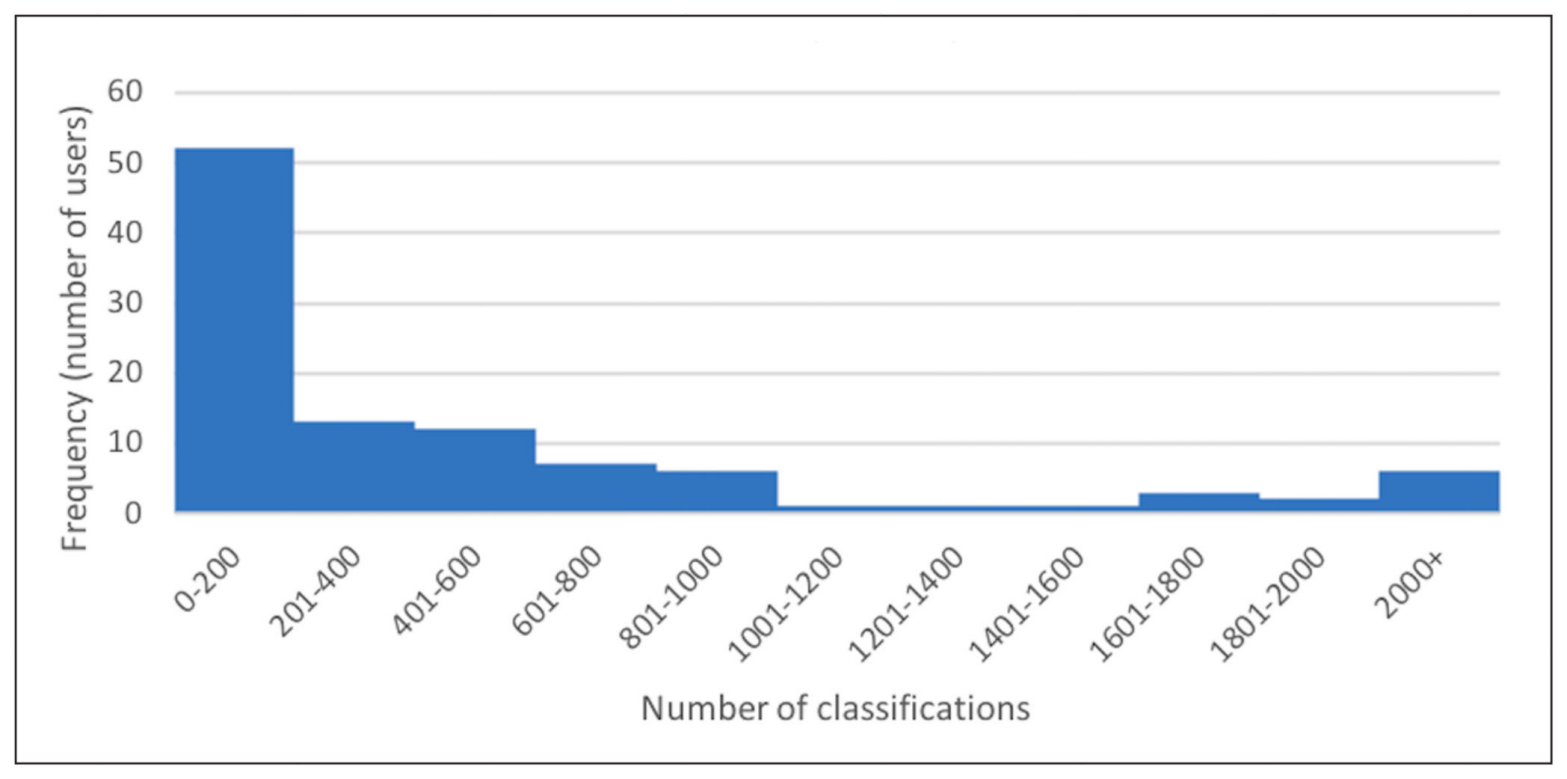
Allocation of Zooniverse classifications amongst participants.

Fifty percent of participants (n = 52) were found to have 200 or fewer classifications over the period of this study. Six participants presented rather unusual behaviour, exhibiting more than 2,000 classifications, and seven were found to have between 1,600 and 2,000 classifications (median = 199). Figure 6 presents the distribution of classifications across participants; the majority contributed few tasks whereas a few participants made the majority of contributions.

Female participants made 35,129 classifications, whereas male participants made 26,221. Participants classifying themselves as “Other” contributed 1,308 classifications (total number of classifications made 62,659). An independent Kruskal-Wallis test showed no statistically significant differences in the number of contributions made between male and female (*p* = .657, NS; p = .05), suggesting that the above number difference is random.

### Project popularity amongst young people

**Figure 7:**
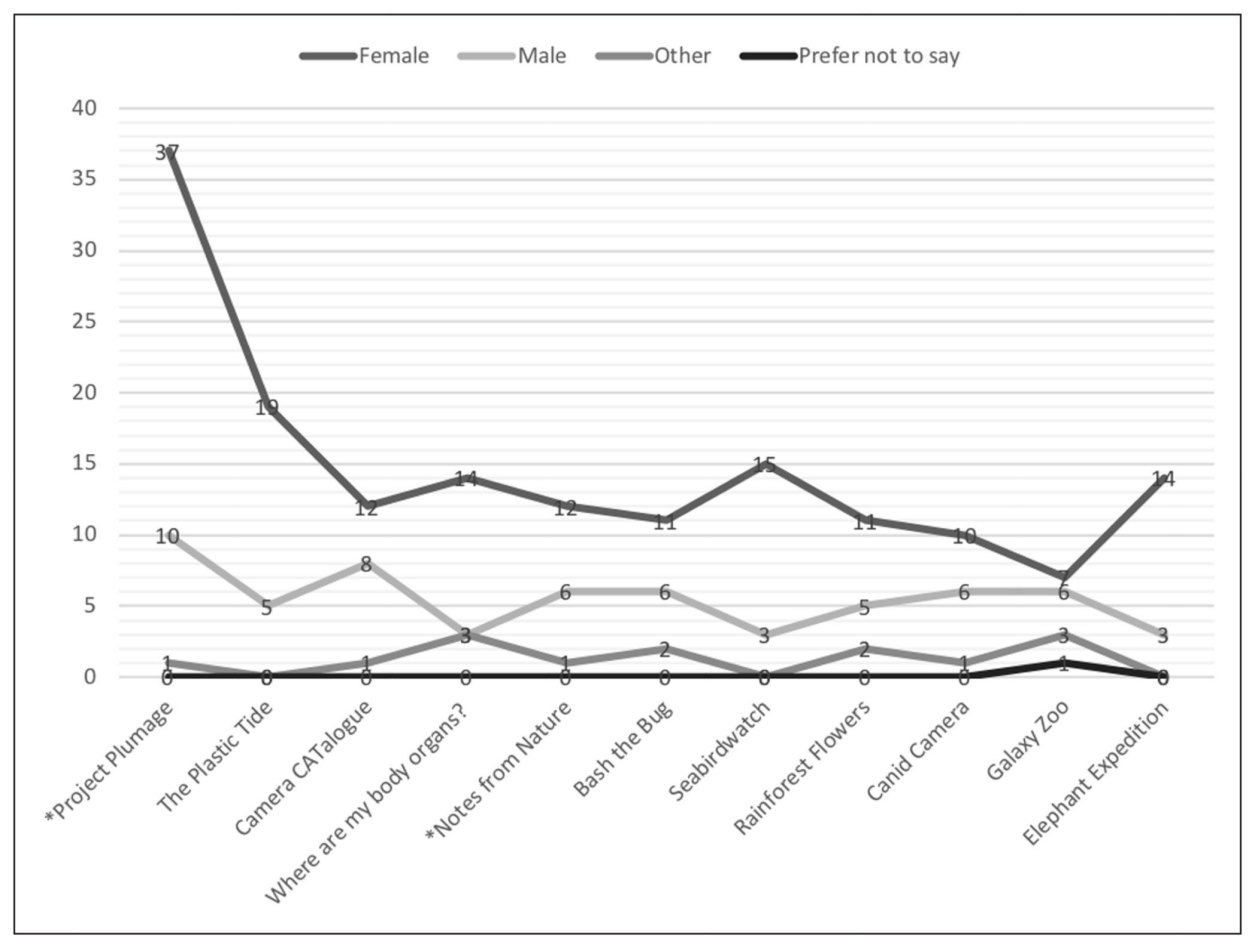
The 11 most popular Zooniverse projects that young people joined analysed by gender.

As expected due to our initial recruitment strategy and the emphasis on a specific NHM project, the most popular Zooniverse project chosen by young people was *Project Plumage* (n = 48). The second and third most popular were projects not explicitly targeted through the project, *Plastic Tide* (n = 24) and *Camera CATalogue* (n = 21). Yet, the *Plastic Tide* was systematically promoted by Zooniverse after its launch in April 2017. *Camera CATalogue* is more likely to reflect young people’s “natural” interests in specific CS projects, in particular an interest in identifying big cats and other wildlife in pictures captured by motion-activated camera traps set around the world. Figure 7 shows the distribution of the most popular projects across gender. Follow-up Kruskal-Wallis independent tests, comparing the distribution of male and female participants across all projects that young people joined, revealed no statistically significant differences for either male (p = .504, NS) or female (p = .555, NS; p < .05), suggesting that males and females participate in similar Zooniverse projects.

In a follow-up analysis, we identified and mapped the most popular projects and their corresponding tasks to the five engagement profiles to explore whether task types relate to specific participation patterns. Types of project tasks were as follows:

(a) Drawing: This task is used to make markings on an image. The various drawing tools include points, lines, rectangles, and ellipses.
(b) Survey: This task asks volunteers to choose from a predefined list of options, e.g., a list of species likely to be found in camera trap images.
(c) Question: This task is used to ask multiple choice questions about an image.
(d) Text: This task allows volunteers to write free text and is used mostly for transcription projects.
(e) Dropdown: This task allows volunteers to set a dropdown list of options, e.g., a list of US States.
(f) Combo: This task allows volunteers to add multiple tasks on the same page, e.g., a text task and a question task shown at the same time.
(g) Slider: This task allows volunteers to set a slider option for choosing between a range of values, e.g., to choose from 1−10 how many animals are in an image.
(h) Shortcut: This task creates a tick box that can be used to skip to the next image, e.g., “There are no animals in this image.”

**Figure 8:**
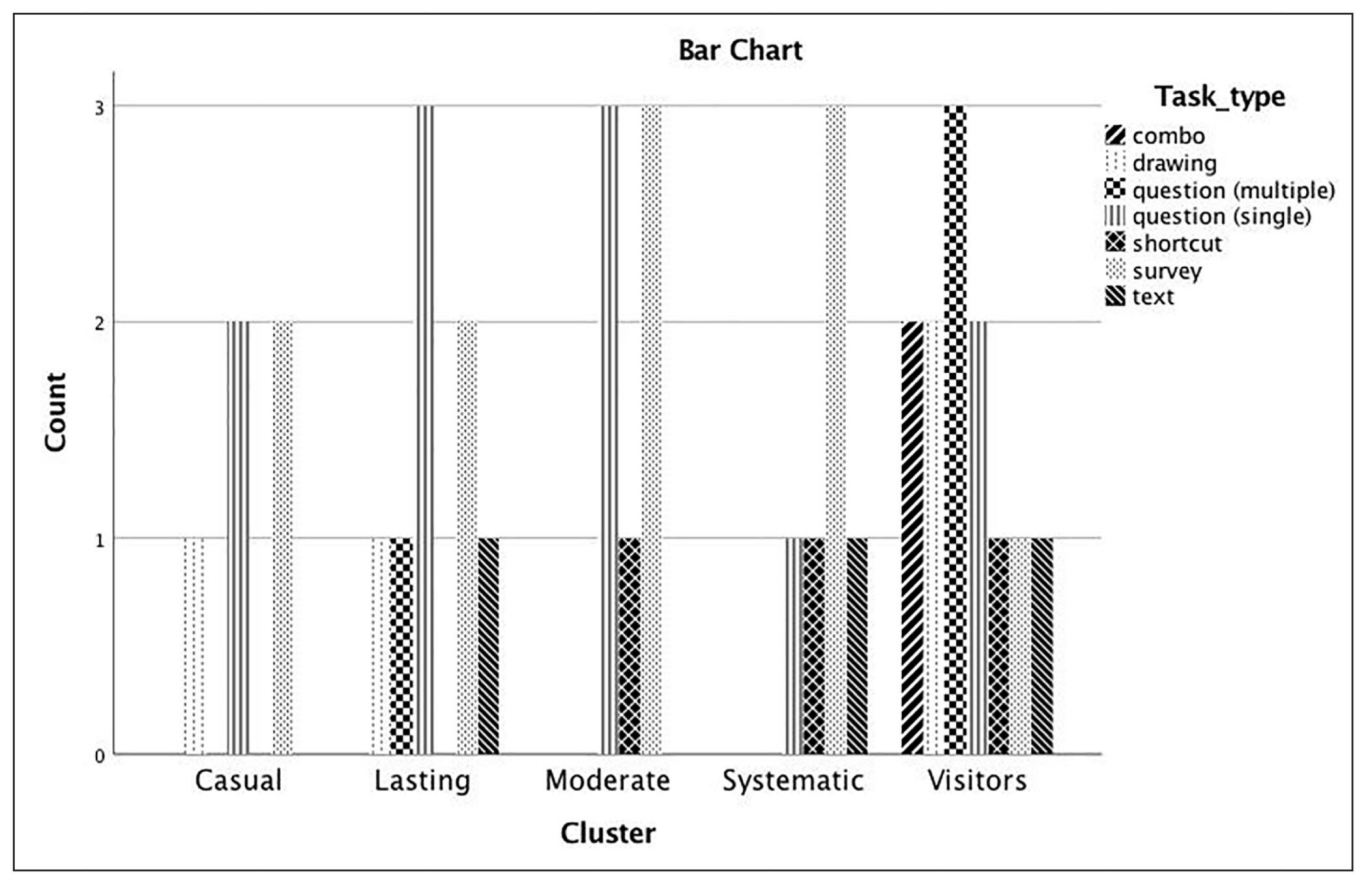
Number of task types within each cluster (most popular projects only).

Figure 8 shows the distribution of task types across the five engagement profiles. The most popular tasks are answering single and multiple choice questions and taking a survey, while combo tasks were chosen only by visitors. Although the graph may point to distinct choices of tasks amongst profiles, a chi-square analysis showed no statistically significant differences between engagement profiles and project tasks of the most popular projects within each cluster (χ^2^(1,38) = 19.847 *p* = 0.705, NS).

### Co-chosen projects

**Figure 9:**
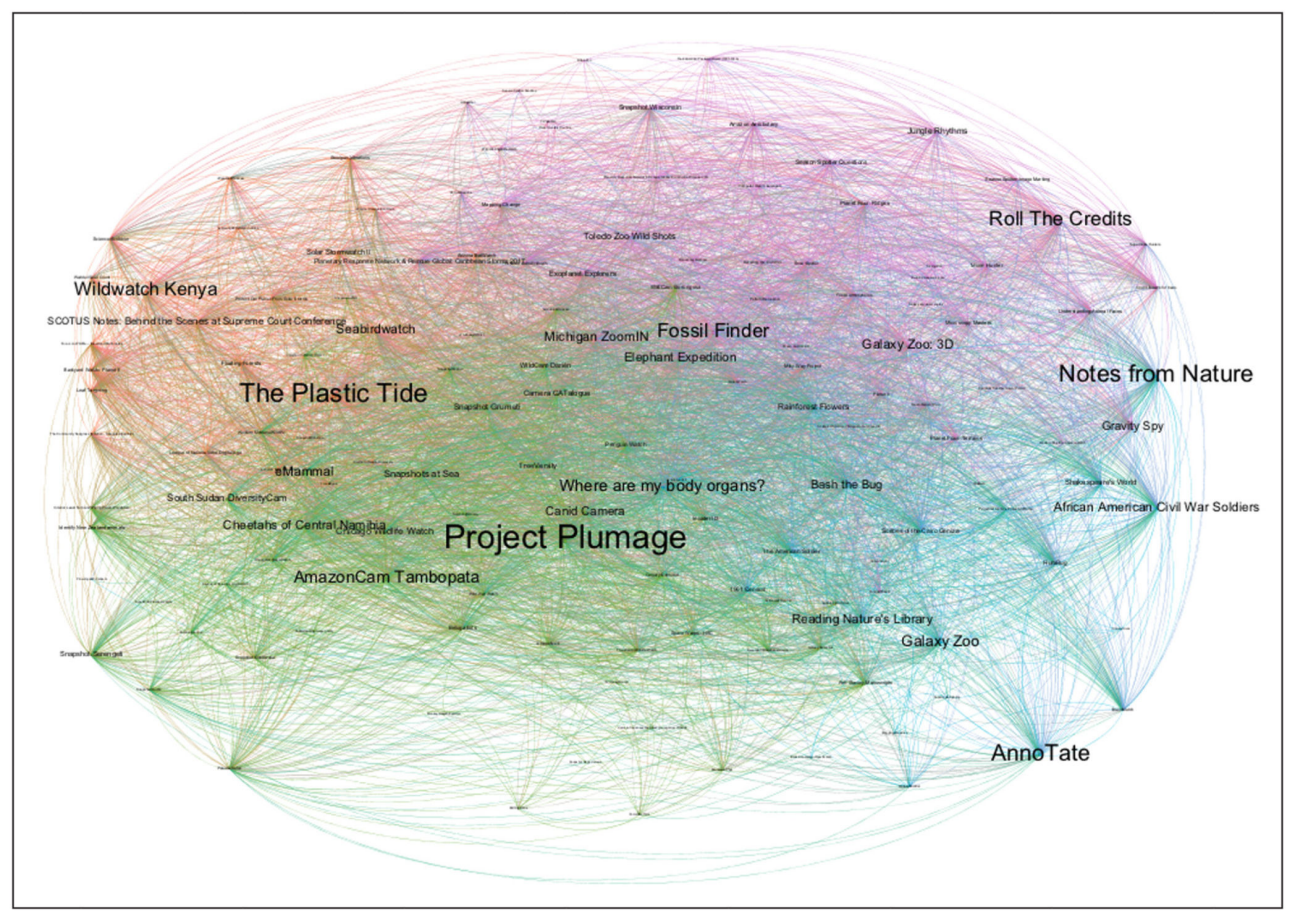
Zooniverse projects chosen together by participants.

The most popular projects chosen together by young people were the *Project Plumage*, the *Plastic Tide*, and *Notes from Nature* (Figure 9). These projects linked indirectly with all other projects, including the less popular ones. The dominance of these three projects is more likely explained by the promotion of *Project Plumage* and *Notes from Nature* in this study, and the fact that *Plastic Tide* was systematically promoted by Zooniverse over the last few years. Regarding the degree of centrality, *Plastic Tide* was the project with the most connections, followed by *Project Plumage* and *Bash the Bag*.

Figure 9 also shows the four sub-communities of projects that tend to be chosen together by young people based on the number of ties between each pair of nodes. The graph suggests that the purple and the green groups, with 35% and 34% graph coverage respectively, were more dominant than the orange and blue ones with 16% and 15% coverage respectively. Examples of projects that belong to the same group are *Plastic Tide* and *Seabird Watch* (in orange) − both projects are UK-based, focus on the environment, and have received major publicity in the UK − and *Bash the Bug* and *Where are my Body Organs* (in blue), which are both biomedical in nature and may suggest that participants had a special interest in biomedicine. Overall, this analysis suggests that targeted publicity and personal interests may explain why young people join specific groups of projects and not others. Given the modularity algorithm result of 0.13, it is noted that there were no dense connections within the groups, yet many connections with projects of other groups. Users co-chose projects from more than one of the coloured groups. A follow-up frequency analysis showed that the majority of users (n = 58) contributed to up to eight projects, while eighteen participants contributed to only one project.

## Discussion

In this exploratory study, we used data analytics and visualisations, clustering, and SNA techniques to examine the participation of 104 young people in the Zooniverse platform. Young people were found to be mainly female, 16−19 years old, with varied patterns of participation. In contrast to adult CS volunteers (Brossard et al. 2005; Price and Lee 2013), the young cohort of volunteers examined in this study was predominantly female. This finding may be explained in a number of ways; it may suggest that within the youth population in particular, CS participants are mainly female, yet a larger sample of participants would be needed to confirm or reject this assumption. Also, it may suggest that the Zooniverse projects under examination, or scientific activities in general, are more appealing or attractive to female volunteers. Follow-up qualitative data analysis such as interviews with volunteers could shed light on this assumption. Overall, this insight aligns well with existing research indicating that globally women (57%) volunteer more than men (43%) (Manchego 2019). Despite the gender imbalance, no gender differences were found in the number of contributions made to Zooniverse projects nor the choice of specific projects, suggesting that young male and female volunteers share similar patterns of activity and tend to participate in similar projects.

The participation of young people in Zooniverse takes a variety of forms and can be classified in five distinct engagement profiles (RQ1):

(a) *Systematic users* are active and visit the platform regularly; this is the profile with the smallest number of users,
(b) *Casual users* have very inconstant visits and are not very active,
(c) *Moderate users* have constant visits, yet they are neither linked to the platform for long nor are they very active,
(d) *Lasting users* are linked to Zooniverse the longest, yet they do not have regular visits and have few active days. This is the profile with the largest concentration of young people, and
(e) *Visitors* contributed to projects one or two days only, yet they are found to be very active during those days.

The proposed classification scheme aligns with the Eveleigh et al. (2014) distinction between “high and low” contributions, and the Ponciano et al. (2014) distinction between “transient” and “regular” levels of engagement. Two of the profiles that we identified, the *Lasting* and *Moderate* profiles, are also found in the adult Zooniverse analysis (Ponciano and Brasileiro 2014), suggesting similar patterns of behaviour between young people and adults. Yet, what this study uniquely contributes is that some young participants demonstrate engagement patterns distinct from those of adults, in particular, the *Systematic* and *Casual* profiles, which are not reported in the analysis of adults (note that these authors excluded Visitors from their analysis). In contrast to adults, some young participants presented systematic patterns of participation, while others were not very active nor did they have constant visits. Overall, young participants were found to be less active and systematic in visiting the platform, yet they remained linked to it for a longer period of time than adults. This insight has important implications for both adapting and designing new online CS programmes; it suggests that young people may still consider themselves participants in a project, and may re-engage in the future, even if they have not been active during a particular period of time.

The *Visitors* profile is one of the largest categories in this study that warrants further examination to understand why young people tend to participate in CS projects few times and then disappear. This is a common pattern of participation in adult CS and other volunteer projects. Also, systematic use is not found to be common amongst young people. Only five participants (4.8%) in this study exhibited this pattern of behaviour. These findings suggest that existing online CS projects may be less attractive to young people or designed in a way that does not meet their needs and interests. Also, a pre-existing interest in science (exhibited in adult CS users, see Brossard et al. 2005) may be required for sustained participation in these projects.

One of the implications of this analysis is the need to identify ways to reinforce young people’s participation in online CS projects, in particular to increase the number of systematic volunteers. An understanding of young people’s motivations for joining and/or quitting CS projects may be a significant starting point. In addition, it is suggested that age-specific clustering analysis can be a powerful tool for understanding CS volunteers and personalising marketing campaigns to the patterns of engagement of different groups of volunteers. For example, an email acknowledging the active participation of a systematic participant and proposing relevant CS projects may be appropriate to the *Systematic* profile. An email acknowledging the infrequent participation of a volunteer, drawing their attention to currently active and relevant projects and opening up communication for reporting possible challenges that can explain low participation may be more appropriate to *Casual* participants or *Visitors*.

The most popular projects amongst young participants (RQ2) were the *Project Plumage* (n = 48), *Plastic Tide* (n = 24), and *Camera CATalogue* (n = 21). Amongst these projects, only *Project Plumage* was actively promoted by the project team. Yet, *Plastic tide* received significant publicity since launched, which may explain its popularity. *Camera CATalogue* is more likely the project reflecting young people’s actual interest in specific CS projects, in particular big cats as captured by motion-activated camera traps. The analysis of the different types of activities (tasks) of the most popular projects within each profile of engagement revealed an overall preference for single/multiple choice and survey questions, yet no differences were found between profiles of engagement, suggesting that the task type is less likely to be related to participation profiles. In terms of the Zooniverse projects that young people choose to participate in (RQ3), SNA showed three projects as being those that are more often chosen together:

a) *Project Plumage* asks people to mark-up different views of bird specimens and help scientists explain colour evolution.
b) *Plastic Tide* is about recognising plastic on images taken by drones and monitoring the marine litter washing up on beaches.
c) *Notes for Nature* is about digitizing information about specimens held in museums.

The choice of these projects may imply an interest in learning more about birds, a concern about marine life and a clean environment, and a general interest in museum collections and natural science. Further analysis showed certain projects that were chosen together by young people. Some of these projects share similarities in content such the *Bash the Bug* and *Where are my Body Organs* − both biomedical projects − an indication that young people joining these may have a special interest in biomedicine. Yet, this is not the case for other projects; a closer examination did not reveal any repeated patterns in content that can explain why these projects are chosen together. The popularity of these projects is more likely explained by the project’s promotional activities for recruiting young people.

Future research should focus on understanding why young people make specific choices of projects as well as which project topics would be of most interest to them. As shown in this analysis, projects that make use of motion-activated cameras to capture wildlife images may be a project type particularly endorsed by young people. Also, it would be useful to identify whether project choices relate or distinguish between the five youth engagement profiles that emerged from this study. In the future, we aim to unpack these trends by interviewing young people from each engagement profile. Qualitative information can inform our understanding of how to design CS projects that are of interest to young people, easy to understand and complete, and motivating enough to sustain systematic participation.

## Conclusions

This paper reported on the first study of its kind that examines young people’s participation (aged 5−19) in online citizen science projects, in particular Zooniverse projects. It is one of the few studies available that made use of learning analytics data and visualisations to capture, analyse, and describe young people’s science engagement and project preferences in informal learning settings. Insights from this study suggest that young people are more likely to be female, 16−19 years old, and less likely to visit online CS projects at regular time intervals and to be very active during their stay (i.e., the systematic engagement profile was found to be the one with the fewer participants). In terms of project preferences, it remains unclear which projects young people tend to participate in due to the targeted recruitment strategy of this project. Also, age-specific clustering analysis is proposed as a suitable tool for understanding participation patterns and tailoring follow-up communication with CS volunteers.

Given that learning is more likely to happen when people participate often and systematically, we need to identify ways to promote young people’s systematic participation and loyalty to online CS, through either the design of new CS projects targeting young people and their interests or redesigning existing programmes in ways that not only spark but also maintain young people’s participation. Despite our understanding of what motivates and sustains adults’ participation in CS programmes, we still do not know whether these insights are applicable to young people. Such projects should also integrate learning objectives in their structure by supporting self-regulated learning processes. Existing studies suggest that variability in project content, scaffolding of the learning process, simple interaction design, and mobile implementation are project features endorsed by young people (Herodotou, Villasclaras-Fernández, and Sharples 2014).

Overall, this study contributed insights about the online behaviour of mainly female, young people, aged 16−19, suggesting that people younger than 16 years old may not be aware or interested in online CS opportunities or that guardians may not be willing to provide the required consent for joining a CS study. It might also be the case that available CS projects are too difficult to understand or to engage younger audiences as they have been designed with an adult population in mind. In the case of Zooniverse, it also may be the case that some users contribute to the platform by themselves or with other family members without being logged in, therefore their contributions cannot be captured and measured.

This study is a significant starting point for understanding and scaffolding young people’s engagement in web-based, informal science learning settings. Online CS projects can be a great opportunity for bringing young people closer to science while also benefiting scientists and helping them understand how science and the scientific process work. Our plans for the next few years are to extend our recruitment strategies to engage more and diverse young audiences, including young people from diverse socioeconomic backgrounds and ages. We also aim to enrich our understanding of engagement with qualitative measures that can unpack why specific engagement patterns are observed, what challenges young people face when joining CS projects, why they select to take part in specific projects, what they learn from participation, and whether these practices have a long-term effect on a person’s identity and future aspirations. Clustering analysis and SNA have been shown to be particularly useful in unveiling trends in online CS including different forms of participation and project choices. These trends can guide us in selecting participants in follow-up studies (e.g., stratified sampling based on the proposed engagement profiles) and developing instruments (e.g., surveys) that build on and question these trends.
